# Gallbladder Tuberculosis Mimicking Gallbladder Carcinoma: A Case Report and Literature Review

**DOI:** 10.1155/2016/3629708

**Published:** 2016-04-21

**Authors:** Yao Liu, Kai Wang, Heng Liu

**Affiliations:** ^1^Department of Hepatobiliary Surgery, Affiliated Hospital of Zunyi Medical College, 149 Dalian Road, Zunyi 563000, China; ^2^Department of Pathology, Affiliated Hospital of Zunyi Medical College, 149 Dalian Road, Zunyi 563000, China; ^3^Department of Radiology, Affiliated Hospital of Zunyi Medical College, 149 Dalian Road, Zunyi 563000, China

## Abstract

Gallbladder tuberculosis (GT) is extremely rare, and it is difficult to differentiate from other gallbladder diseases, such as gallbladder carcinoma and Xanthogranulomatous Cholecystitis. A correct preoperative diagnosis of GT is difficult. The final diagnosis is usually made postoperatively according to surgical biopsy. Here, we report a case of a patient who underwent surgery with the preoperative diagnosis of gallbladder carcinoma. We reviewed the literature and present the process of differential diagnosis between two or more conditions that share similar signs or symptoms.

## 1. Introduction

Gallbladder tuberculosis (GT) is an extremely rare disease entity. Gallbladder is mostly infected with hematogenous tuberculosis or other intra-abdominal tuberculoses [1, 2]. A correct preoperative diagnosis of GT is unusual, which is frequently difficult in differentiating from other gallbladder diseases. Since the first case of GT was reported in 1870 by Gaucher [3], few cases have been reported in the literature. Here, we report a case of a patient who underwent surgery with the preoperative diagnosis of gallbladder carcinoma after computed tomography (CT) scan. We also highlight the importance of differential diagnosis between two or more conditions that share similar signs or symptoms.

## 2. Case Report

**Figure 1:**
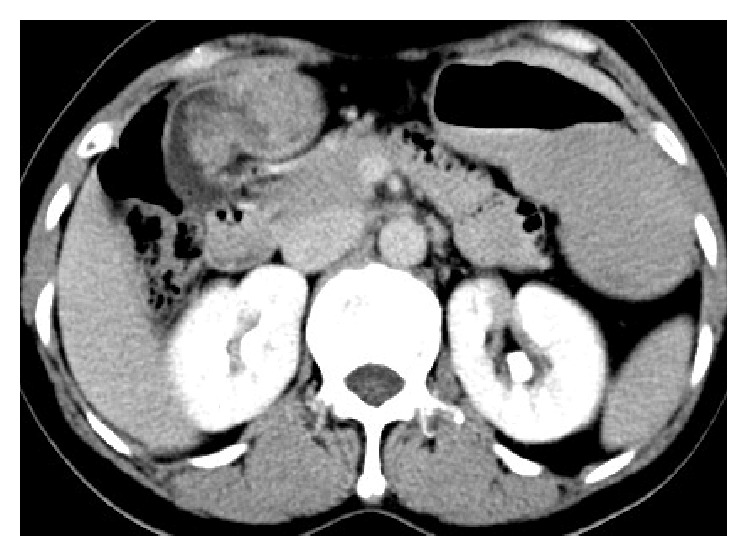
CT scan reveals a thickening gallbladder wall and heterogeneous enhancement of the gallbladder wall, with the interface between the gallbladder wall and the liver not apparent.

A 68-year-old male patient was admitted to our hospital with chief complaints of recurrent pain in right hypochondrium, loss of appetite, and dyspepsia for the past 1 month. No fever, weight loss, or night sweating was reported. The patient denied any history of tuberculosis or hepatitis. There was no history of jaundice. The general and abdominal examinations revealed nothing significant. Laboratory investigations were normal. No abnormality was found at chest X-ray. At abdominal ultrasound, a gallbladder mass was observed, and the gallbladder was distended with multiple gallstones with associated cholecystitis. Preoperative ESR was 30 mm/h. Contrast-enhanced CT scan revealed a 5 mm thick gallbladder wall, and the interface between liver and gallbladder was not obvious, possibly indicating that gallbladder lesions had infiltrated into the liver parenchyma (Figure 1). Therefore, a diagnosis of gallbladder carcinoma was considered, and the patient underwent a subcostal laparotomy. Intraoperation, we found that the gallbladder size was around 9 cm × 5 cm × 4 cm, and there was edema in gallbladder wall. A stone was incarcerated at the neck, and gallbladder was filled with pus.

**Figure 2:**
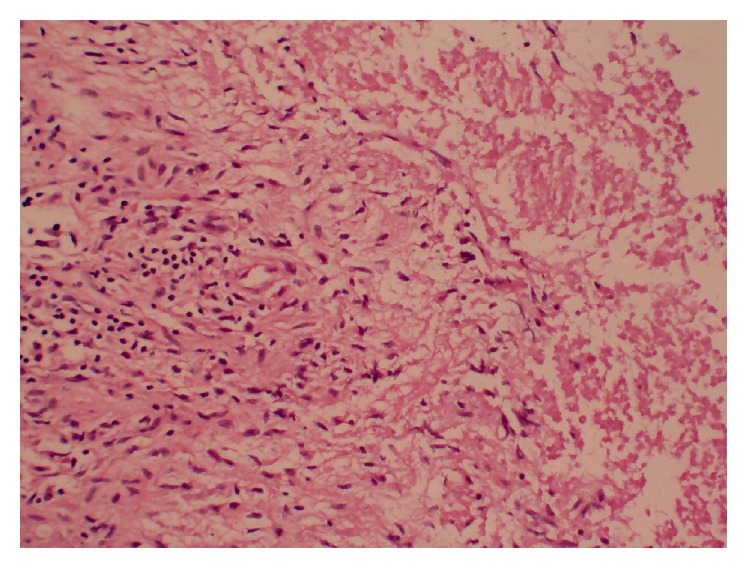
Microscopical view shows epithelioid granuloma along with typical Langhans giant cells (HE, original magnification ×20).

The gallbladder was resected and sent for intraoperative frozen-section examination, and report indicated that no sign of malignancy was found. The definitive histopathological examination (HPE) of gallbladder reported gallbladder tuberculosis (Figure 2). Microscopical view shows epithelioid granuloma along with typical Langhans giant cells. The Mantoux tuberculin skin test was positive, and no other findings suggestive of tubercular infection were found. Therefore, the patient received antitubercular therapy for 6 months.

## 3. Outcome and Follow-Up

The patient has been gradually relieved of all symptoms since cholecystectomy and was well at 6-month follow-up.

## 4. Discussion

Gallbladder tuberculosis (GT), especially the isolated GT, is an exceedingly rare disease entity [4]. GT often occurs along with other intra-abdomen tuberculoses, which reach the gallbladder via the lymphatics or bloodstream, and GT mostly happens in women over the age of 30 [2, 5]. Gallbladder is not a susceptible organ to tuberculosis, possibly owing to the inhibitory effect of bile. Cholelithiasis and cystic duct obstruction are deemed to be important factors in development of GT [6].

Although patients infected with GT may present with a series of symptoms such as abdominal pain, jaundice, weight loss, vomiting, and abdominal mass, pain in right hypochondrium and abdominal mass may be main clinical manifestations. Around 70% of GT cases are accompanied by gallstones [7].

A correct preoperative diagnosis of GT is difficult. Therefore, differential diagnosis from other gallbladder diseases is important. For patients who have been infected with GT, an increased ESR and anemia, along with positive Mantoux tuberculin skin test, would be detected in laboratory tests. For gallbladder carcinoma (GC), the most common symptoms are pain (76%), weight loss (39%), jaundice (38%), and anorexia (32%) [8, 9]. Besides, serum CA19-9 is usually elevated. By contrast, moderate elevation of CA19-9 was often detected in patients with Xanthogranulomatous Cholecystitis (XGC), so serum level of CA19-9 is not helpful for distinguishing XGC from GC [10, 11]. There are no typical symptoms or clinical signs for XGC, and clinical manifestation of XGC is similar to that of cholecystitis [12, 13].

In aspect of radiology, CT scan or magnetic resonance imaging (MRI) is necessary for differential diagnosis. Among GC patients, intraluminal polypoid mass or invasion to adjacent organ and vessel can be easily used to differentiate from gallbladder tuberculosis and XGC [10, 14]. Besides, thickening gallbladder wall can be frequently observed in early-stage GC patients [15]. It has been reported that a continuous mucosal line, gallstone, pericholecystic infiltration, diffuse thickening wall, and hypoattenuated intramural nodule were significant CT manifestations of XGC [16]. Characteristically, enhanced gallbladder wall was more commonly observed in GC than XGC, and enhancement pattern also seems different [17, 18].

Xu et al. revealed that there are three types of CT morphology features for GT diagnosis [19]. The micronodular type is characterized by polypoid or micronodular lesion, which shows homogeneously enhanced gallbladder wall in contrast-enhanced CT scan. The most common form of GT is the thickened-wall type, which can be frequently misdiagnosed as GC or cholecystitis [20]. The wall is mostly thickened, uniform, and diffuse. Besides, the edema “halo” in GT patients can be observed on CT scan unlike that in GC patients [21, 22]. The CT manifestation of the mass-forming type is similar to GC, which shows flecked calcification of gallbladder wall [23]. Totally, a tissue mass with multicentre necrosis or with multiple calcification on enhanced CT scan would be useful to distinguish gallbladder tuberculosis from XGC and gallbladder carcinoma.

In conclusion, CT manifestation, combined with clinical symptoms, might be an available approach to diagnose GT. The final diagnosis of GT relies on the histopathological examination of resected specimen.
